# The Pivotal Roles of US3 Protein in Cell-to-Cell Spread and Virion Nuclear Egress of Duck Plague Virus

**DOI:** 10.1038/s41598-020-64190-2

**Published:** 2020-04-28

**Authors:** Liyao Deng, Mingshu Wang, Anchun Cheng, Qiao Yang, Ying Wu, Renyong Jia, Shun Chen, Dekang Zhu, Mafeng Liu, Xinxin Zhao, Shaqiu Zhang, Juan Huang, Xumin Ou, Sai Mao, Ling Zhang, Yunya Liu, Yanling Yu, Bin Tian, Leichang Pan, Mujeeb Ur Rehman, Xiaoyue Chen

**Affiliations:** 1 Institute of Preventive Veterinary Medicine, Sichuan Agricultural University, Wenjiang, 611130 People’s Republic of China; 2 Avian Disease Research Center, College of Veterinary Medicine of Sichuan Agricultural University, Wenjiang, 611130 People’s Republic of China; 3 Key Laboratory of Animal Disease and Human Health of Sichuan Province, Sichuan Agricultural University, Wenjiang, 611130 People’s Republic of China

**Keywords:** Herpes virus, DNA synthesis

## Abstract

The duck plague virus (DPV) US3 protein, a homolog of the herpes simplex virus-1 (HSV-1) US3 protein that is reported to be critical for viral replication, has been minimally studied. Therefore, to investigate the function of the DPV US3 protein, we used scarless Red recombination technology based on an infectious bacterial artificial chromosome (BAC) containing the DPV Chinese virulent strain (CHv) genome and successfully constructed and rescued a US3-deleted mutant and the corresponding revertant virus (BAC-CHv-ΔUS3 and BAC-CHv-ΔUS3R, respectively). For viral growth characteristics, compared to the parental and revertant viruses, the US3-deleted mutant showed an approximately 100-fold reduction in viral titers but no significant reduction in genome copies, indicating that the US3-deleted mutant exhibited decreased viral replication but not decreased viral DNA generation. In addition, the US3-deleted mutant formed viral plaques that were 33% smaller on average than those formed by the parental and revertant viruses, demonstrating that US3 protein affected the viral cell-to-cell spread of DPV. Finally, the results of electron microscopy showed that the deletion of US3 resulted in a large number of virions accumulating in the nucleus and perinuclear space, thus blocking virion nuclear egress. In this study, we found that the DPV US3 protein played pivotal roles in viral replication by promoting viral cell-to-cell spread and virion nuclear egress, which may provide some references for research on the function of the DPV US3 protein.

## Introduction

Duck plague, also known as duck viral enteritis (DVE), is an acute, febrile, septic disease that appears in waterfowl and is caused by duck plague virus (DPV). Duck plague spreads rapidly with high morbidity and mortality, resulting in significant economic losses in the avian industry. DPV belongs to the *Alphaherpesvirinae* subfamily and contains a double-stranded helical DNA consisting of a unique long (UL) region, a unique short (US) region, a unique short internal repeat (IRS) region and a unique short terminal repeat (TRS) region, thus forming the UL-IRS-US-TRS structure of the viral genome^1–3^.

The characteristics of some genes of DPV have been reported. Herpesvirus genes are classified into immediate-early (IE), early (E) and late (L) genes according to their order of gene expression and thereby play different roles in viral replication. The kinetic classes of many DPV genes have been identified. DPV UL54, an IE gene, can shuttle between the nucleus and cytoplasm to regulate viral replication, and the recombinant UL54-deleted virus produced smaller viral plaque sizes and lower viral genome copies than the parental virus^4–7^. DPV UL13 is an E gene and localizes to the nucleus and cytoplasm, and knocking out UL13 impaired viral replication^8^. Most DPV genes, including US2^9^, US5^10^, US10^11^, UL16^12^, UL35^13^, UL41^14^, UL53^15^, and UL55^16^, are classified as L genes. To date, only a few proteins encoded by DPV apart from UL54 and UL13 have been studied in mutant viruses. Both the gJ (US5) and US10 proteins of DPV affect viral replication as proven using recombinant viruses. The gJ deletion virus of DPV reduced cell-to-cell spread, compromised virion assembly and envelopment, and increased apoptosis in infected duck embryonic fibroblast (DEF) cells^10,17^. Deletion of US10 decreased viral titers but did not change the genome copies and the transcriptional levels of immune-related genes, e.g., toll-like receptor 3 (TLR3), myxovirus resistant (Mx), oligoadenylate synthetases-like (OASL), interleukin (IL) -4, IL-6 and IL-10^18^. However, not all proteins encoded by DPV are essential for viral replication, e.g., UL55. The growth kinetics, plaque morphology and viral titers of a UL55-deleted virus were similar to those of the parental virus, suggesting that UL55 is dispensable for DPV replication^19^.

Alpha-herpesvirus US3 protein has been reported to be a serine/threonine kinase that phosphorylates a variety of proteins, including the viral proteins UL31, UL34, UL47 and gB^20–22^, and the host proteins Lamin A/C, p65, IRF3, group A p21-activated kinases (PAKs) and Bad^23–27^, a proapoptotic protein. The effect of the herpes simplex virus-1 (HSV-1) US3 protein on virion nuclear egress is related to kinase activity. Both US3 deletion and US3 kinase-dead mutations of HSV-1 caused virion accumulation in the perinuclear space, which was regulated by the phosphorylation of UL31, UL34, UL47, gB and Lamin A/C through US3 protein^28–31^. Viral cell-to-cell spread facilitates viral replication by enabling a virus to evade host immune surveillance. The pseudorabies virus (PRV) US3 protein was first reported to generate long actin- and microtubule-containing cell projections that allowed the virus to move into neighboring cells. PAKs, the key regulators in Rho GTPase signaling pathways, played a pivotal role in the US3-mediated formation of cell projections and were bound to and phosphorylated by US3^26,32^. In addition, US3 protein also regulates the innate immune response and apoptosis to promote viral replication by phosphorylating corresponding substrates^33,34^.

The DPV US3 protein is predicted to be a serine/threonine protein kinase and a homolog of the HSV-1 US3 protein^3^. Due to the extensive availability of phosphorylation substrates and the powerful functions of US3 protein encoded by other alpha herpesviruses, studying the DPV US3 protein is essential for understanding DPV pathogenesis. In this study, to clarify the role of the US3 protein in DPV replication, we constructed a US3-deleted mutant and its revertant virus using a scarless Red recombination system and detected their biological characteristics. The results demonstrated that the US3-deleted mutant exhibited significantly reduced viral titers and plaque sizes. Electron microscopy analysis indicated that the US3-deleted mutant displayed accumulation of a large number of virions in the nucleus and perinuclear space, preventing nucleocapsids from undergoing further assembly and maturation. These data are the first to show that the DPV US3 protein affects viral replication by regulating viral cell-to-cell spread and virion nuclear egress, which will provide some references for research on the function of US3 protein and the prevention and control of DPV.

## Results

### Construction and identification of recombinant pBACs

**Figure 1:**
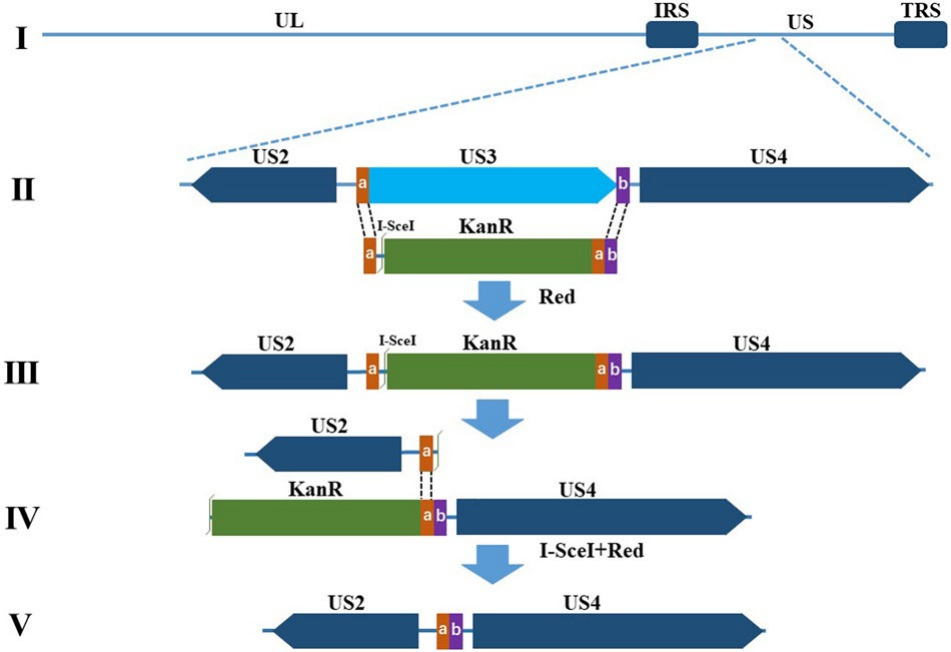
Schematic diagram of pBAC-CHv-ΔUS3 construction. (**I**) The DPV CHv genome consists of the UL, IRS, US and TRS regions. (**II**) The US3 gene is replaced with the KanR cassette in the first Red recombination through the 40-bp homology arms (sequences **a**,**b**). (**III**) The intermediate product after the first Red recombination is shown. (**IV**) The KanR cassette is removed in the second Red recombination through I-SceI and a 40-bp sequence duplication (sequence **a**). (**V**) The genome of pBAC-CHv-ΔUS3 is shown.

**Figure 2:**
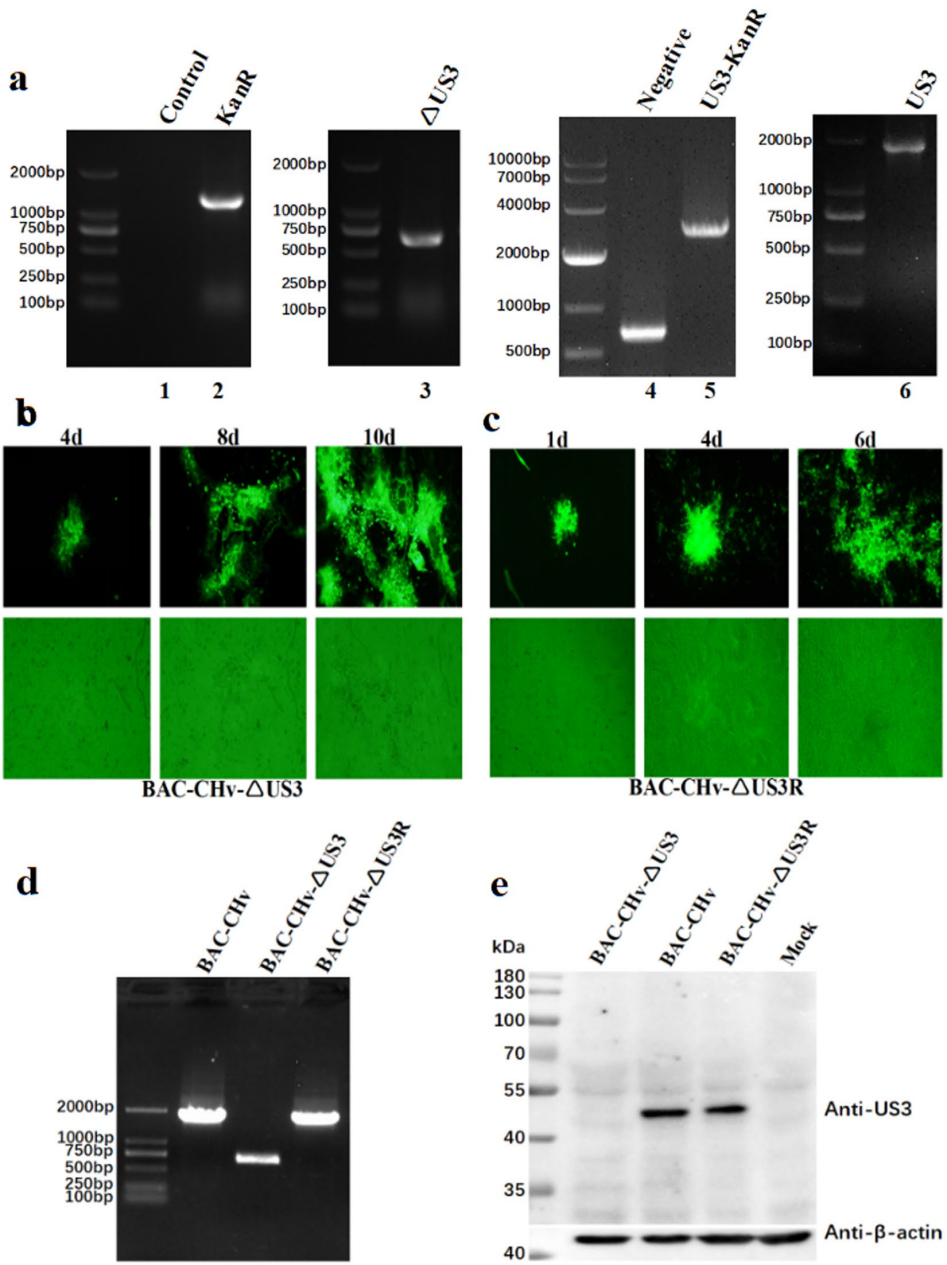
Construction and identification of recombinant viruses. (**a**) Identification of recombinant pBACs by PCR analysis. The DNA bands of KanR (lane 2), a US3 flanking sequence (lane 3), US3 + KanR (lane 5) and US3 (lane 6) were amplified. No template was used in the control group (lane 1), and lane 4 was the negative result. (**b**) Rescue of the US3-deleted mutant. Green fluorescent plaques and the corresponding cells were observed at 4, 8 and 10 d after transfection. (**c**) Rescue of the revertant virus. Green fluorescent plaques and the corresponding cells were observed at 1, 4 and 6 d after transfection. (**d**) Identification of recombinant viruses by PCR analysis. Viral DNA was extracted and amplified by PCR. (**e**) Western blot analysis of US3 protein expression of recombinant viruses. Lysates of cells infected with recombinant viruses were subjected to western blotting, and an anti-US3 polyclonal antibody was used to detect the protein expression of US3. β-actin was used as a control.

A bacterial artificial chromosome (BAC) plasmid containing the DPV Chinese virulent strain (CHv) genome (pBAC-CHv) was constructed and transfected into *Escherichia coli* GS1783 in our laboratory^35^. To examine the function of the DPV US3 protein, we used a scarless Red recombination system in *E. coli* GS1783, which allowed scarless-mediated generation of deletions and insertions of small and large sequences in BAC clones^36^, and constructed a US3-deleted mutant BAC (pBAC-CHv-ΔUS3) derived from pBAC-CHv as described in the Materials and Methods. The US3 open reading frame (ORF) was entirely removed from the genome of pBAC-CHv (Fig. 1). To more accurately determine US3 protein’s function, a US3-revertant mutant BAC (pBAC-CHv-ΔUS3R) was also constructed. The constructed recombinant pBACs were identified by PCR analysis. As expected, DNA bands of approximately 1,400 bp (lane 2), 600 bp (lane 3), 3,000 bp (lane 5) and 1,800 bp (lane 6) were amplified separately, and the corresponding products were KanR, a US3 flanking sequence, US3-KanR and US3, respectively (Fig. 2a). No band was detected in the control group that had no template (Fig. 2a, lane 1) to ensure the primers’ specificity. Lane 4 (Fig. 2a) was the negative result.

### Rescue and confirmation of recombinant viruses

The pBAC-CHv-ΔUS3 and pBAC-CHv-ΔUS3R plasmids were extracted and transfected into DEF cells. On the 4^th^ day after pBAC-CHv-ΔUS3 transfection, green fluorescent plaques with matching cytopathic effects were first observed in the DEF cells. On the 10^th^ day after transfection, high levels of green fluorescent plaques were noted (Fig. 2b). Then, the DEF cells were collected and passaged in new cells at least 3 times. Green fluorescent plaques were steadily generated in the new DEF cells, indicating that the US3-deleted mutant (BAC-CHv-ΔUS3) was rescued successfully. Similarly, the revertant virus (BAC-CHv-ΔUS3R) was also rescued and harvested successfully. However, green fluorescent plaques were first observed on the 1^st^ day after pBAC-CHv-ΔUS3R transfection, and a large number of green fluorescent plaques with serious cytopathic effects were generated on the 6^th^ day after transfection (Fig. 2c), resulting in cells that could not be cultured for up to 10 days and had to be collected in advance. Because the green fluorescent plaques of the US3-deleted mutant were first generated later than those of the revertant virus after transfection, we speculated that US3 deletion might impair DPV replication.

To identify the rescued viruses, viral DNA was extracted from DEF cells infected with the US3-deleted or revertant virus, and PCR analysis was performed. DNA bands of approximately 600 bp (BAC-CHv-ΔUS3) and 1,800 bp (BAC-CHv-ΔUS3R) were amplified as expected (Fig. 2d), and viral DNA of the parental virus (BAC-CHv) was used as a positive control. Western blot analysis of the expression of US3 protein in infected cells was also performed for further confirmation. As shown in Fig. 2e, US3 protein expression was detected only in the parental virus- and revertant virus-infected cells and not in the US3-deleted mutant-infected cells, indicating that the rescued viruses were available for subsequent experiments.

### Effect of the DPV US3 protein on viral growth kinetics

**Figure 3:**
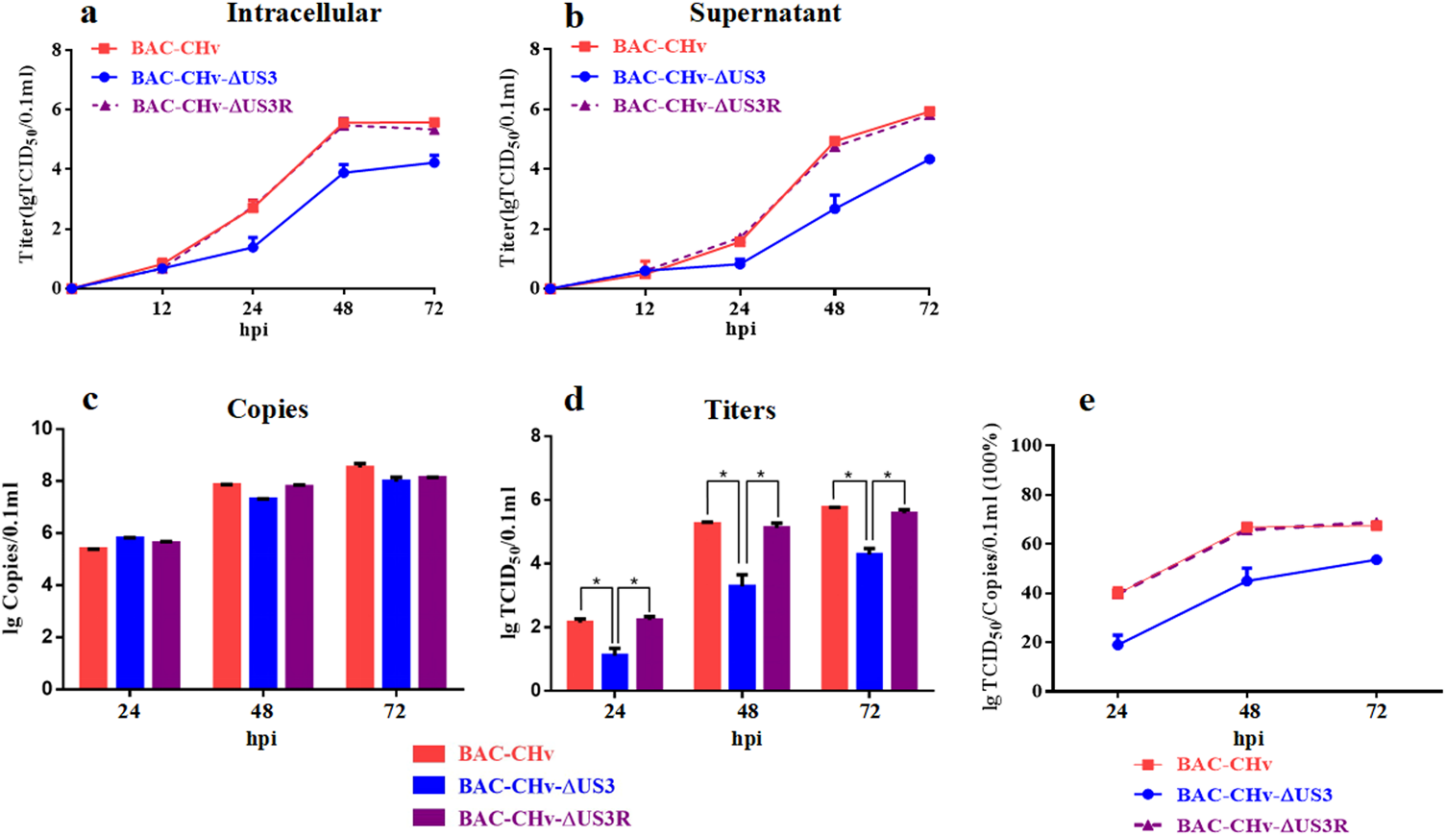
Determination of viral titers and genome copies in growth kinetics. DEF cells in 24-well plates were infected with 0.05 MOI of BAC-CHv, BAC-CHv-ΔUS3 or BAC-CHv-ΔUS3R. Samples were collected at the indicated time points, and viral titers and genome copies were determined. The data were presented as the mean ± standard deviation (SD) of three independent experiments. (**a**) Intracellular viral titers. (**b**) Supernatant viral titers. (**c**) Viral genome copies in total cells. (**d**) Viral titers in total cells. (**e**) The efficiency of infectious virion formation (Titers/Genome copies).

To investigate whether US3 protein played a role in DPV replication, viral growth kinetics were detected. DEF cells infected with 0.05 multiplicity of infection (MOI) of BAC-CHv, BAC-CHv-ΔUS3 and BAC-CHv-ΔUS3R were harvested at several time points, and intracellular viral titers and supernatant viral titers were determined. As shown in Fig. 3a, all incubated viruses entered cells and initiated viral replication, and the intracellular viral titers of the three strains did not significantly differ at the early stage of infection. At 12 hours post infection (hpi), intracellular viral titers were rapidly increased until 48 hpi, suggesting a major replication stage for all strains during this time period. Subsequently, a slight fluctuation in titers was observed from 48 to 72 hpi. Concomitantly, supernatant viral titers slowly increased until 24 hpi and then increased substantially from 24 to 72 hpi (Fig. 3b), indicating that major viral release occurred between 24 and 72 hpi. Regardless of the intracellular or supernatant source, the viral titers of the US3-deleted mutant were significantly lower than those of the parental virus from 12 hpi, with an approximately 100-fold reduction observed between 48 and 72 hpi, and the growth efficiency was restored to the level of the parental virus by reintroducing US3 in the revertant virus (Fig. 3a,b). These results indicated that US3 deletion impaired DPV replication.

A previous study showed that the DPV UL54-deleted mutant had a viral growth defect that caused relatively low production of viral genome copies^6^. Therefore, to detect whether US3 deletion impaired DPV DNA generation, viral genome copies of the three strains were detected by qPCR. As shown in Fig. 3c, the number of viral genome copies of the US3-deleted mutant was slightly lower than those of the parental and revertant viruses at 48 hpi and 72 hpi, but this result was negligible compared with the finding of significantly reduced viral titers (Fig. 3d), suggesting that US3 protein may not affect viral DNA generation. In addition, the efficiency of infectious virion formation (Titers/Genome copies) of the US3-deleted mutant was obviously lower than that of the parental and revertant viruses (Fig. 3e), indicating that US3 protein might play a role in the maturation of infectious virions.

### Effect of the DPV US3 protein on viral cell-to-cell spread

**Figure 4:**
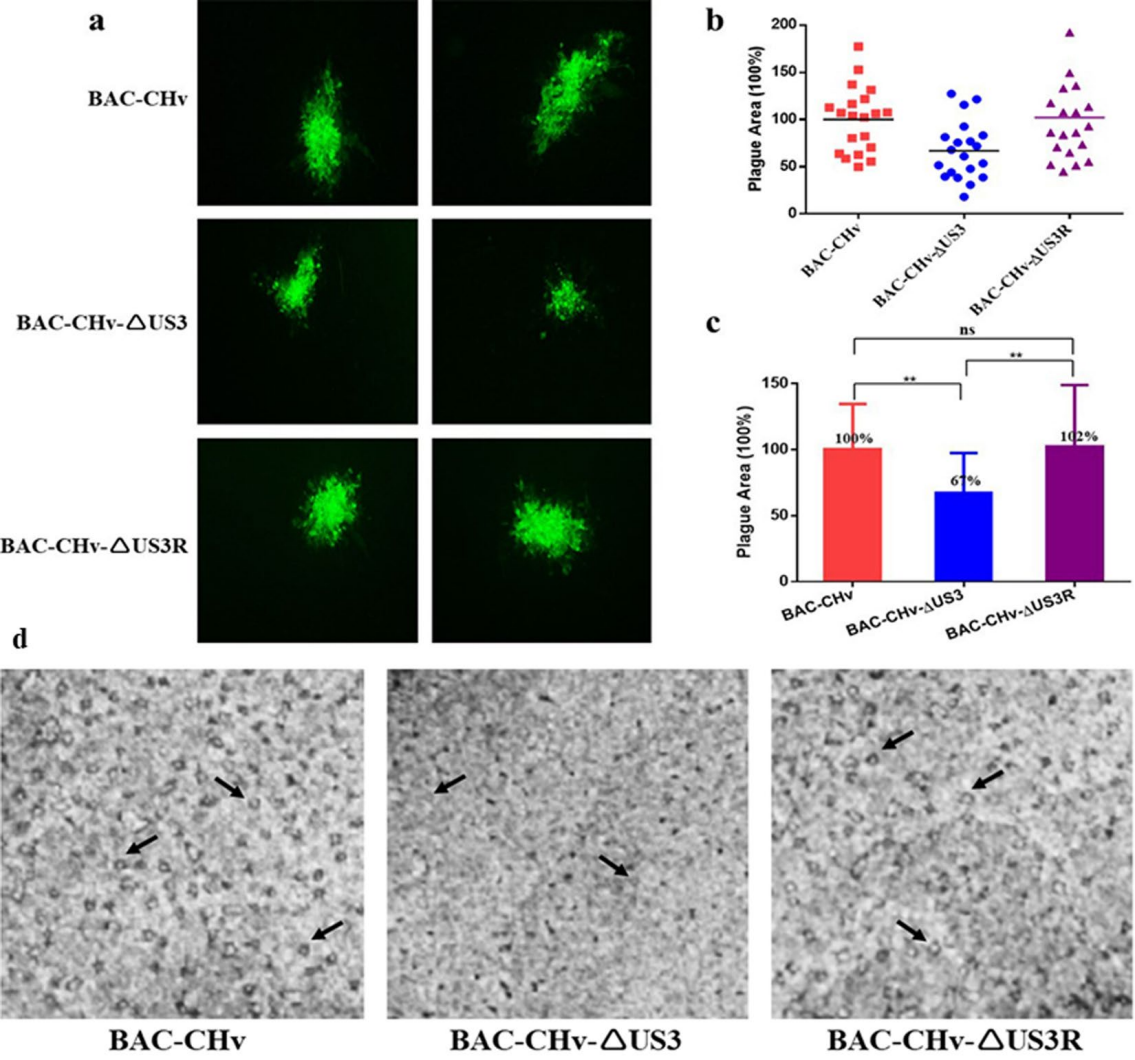
Determination of cell-to-cell spread by plaque assays. DEF cells in 6-well plates were infected with 0.001 MOI of BAC-CHv, BAC-CHv-ΔUS3 or BAC-CHv-ΔUS3R. After incubation at 37 °C for 2 h, the infected cells were covered with 1% methylcellulose and cultured. (**a**) Green fluorescent plaques produced by the US3-deleted, parental and revertant viruses. (**b**) and (**c**) Statistical analysis of twenty different randomly selected viral green fluorescent plaques. (**d**) Images of viral plaques after 1.5% crystal violet staining. The black arrows indicate viral plaques.

The PRV US3 protein has been reported to generate long actin- and microtubule-containing cell projections to promote viral cell-to-cell spread^32^. Therefore, we performed plaque morphology assays to explore the effects of the DPV US3 protein on transmission between adjacent cells. As shown in Fig. 4a, the US3-deleted mutant produced smaller green fluorescent plaques than the parental and revertant viruses. To better assess green fluorescent plaque sizes, 20 randomly selected green fluorescent plaques generated by the three strains were statistically analyzed. The plaque sizes of the parental virus were set to 100%. As shown in Fig. 4b,c, on average, the plaque sizes produced by the US3-deleted mutant were 67%, which is approximately 33% smaller than those produced by the parental virus. As expected, the plaque sizes were restored for the revertant virus, which had an average plaque sizes of 102%, confirming that the US3-deleted mutant produced smaller plaques than the parental strain. Since green fluorescent plaques were not exactly equal to viral plaques, we also detected viral plaques using crystal violet staining. As shown in Fig. 4d, the US3-deleted mutant still produced smaller viral plaques than the parental and revertant viruses. These data indicated that the DPV US3 protein influenced viral cell-to-cell spread, coinciding with the significant reduction in viral titers caused by the deletion of US3.

### Effect of the DPV US3 protein on virion nuclear egress

**Figure 5:**
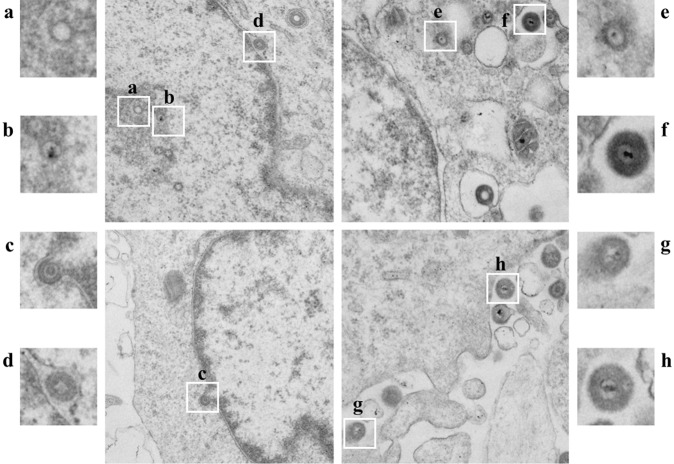
The virion maturation processes of DPV. DEF cells were infected with 5 MOI of BAC-CHv and examined by electron microscopy analysis. The white boxed areas in the central images are enlarged in the insets on the right and left. (**a**) Capsid formation. (**b**) DNA encapsidation in the nucleus. (**c**) Nucleocapsid egress from the nucleus. (**d**) Primary enveloped virion formation in the perinuclear space. (**e**) De-envelopment with naked nucleocapsids in the cytoplasm. (**f**) Secondary envelopment by wrapping with the Golgi membrane and mature virions present in transport vesicles. (**g**) and (**h**) Mature virion exocytosis.

**Figure 6:**
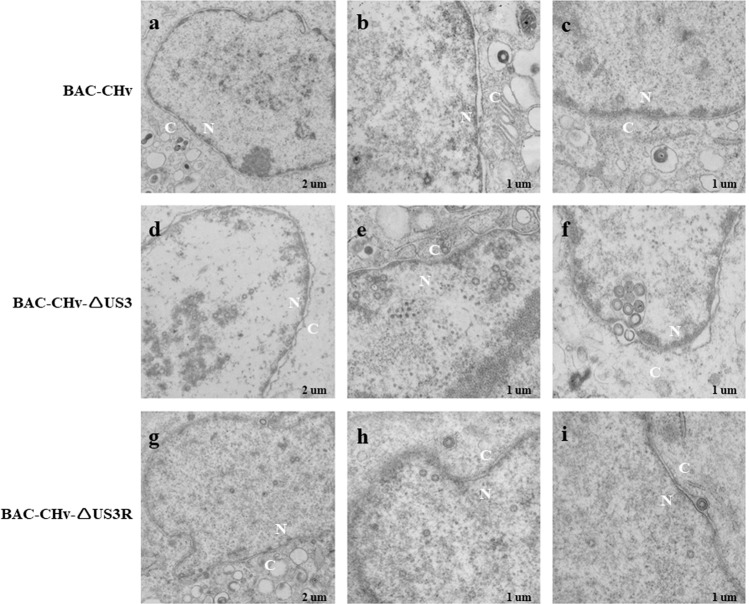
Electron microscopy analysis of DEF cells infected with BAC-CHv, BAC-CHv-ΔUS3 or BAC-CHv-ΔUS3R. (**a–c**) BAC-CHv. (**d–f**) BAC-CHv-ΔUS3. (**g–i**) BAC-CHv-ΔUS3R. The nucleus (**N**) and cytoplasm (**C**) are marked.

The above data showed that the efficiency of infectious virion formation of the US3-deleted mutant was lower than those of the parental and revertant viruses (Fig. 3e). Therefore, to determine the effect of US3 protein on the maturation of infectious virions, we investigated viral morphogenesis by transmission electron microscopy in DEF cells infected with BAC-CHv, BAC-CHv-ΔUS3 or BAC-CHv-ΔUS3R. Figure 5 shows the life cycle of the parental virus, and we identified the complete virion maturation processes of DPV, which included: (1) capsid formation (Fig. 5a) and DNA encapsidation in the nucleus (Fig. 5b), (2) nucleocapsid egress from the nucleus (Fig. 5c), (3) primary enveloped virion formation in the perinuclear space (Fig. 5d), (4) de-envelopment with naked nucleocapsids in the cytoplasm (Fig. 5e), (5) secondary envelopment by wrapping with the Golgi membrane and mature virions present in transport vesicles (Fig. 5f), and (6) mature virion exocytosis (Fig. 5g,h). The US3-deleted virus- and revertant virus-infected cells exhibited the same virion maturation processes as the parental virus (data not shown).

Compared with the parental virus-infected cells, where only a small amount of virions were present in the nucleus (Fig. 6a,b) and few primary enveloped virions were observed in the perinuclear space (Fig. 6c), the US3-deleted mutant-infected DEF cells exhibited extensive virion accumulation in the nucleus (Fig. 6d,e), and a vesicle-like structure wrapped eight primary enveloped virions in the perinuclear space (Fig. 6f). The vesicle-like structure observed in the US3-deleted mutant-infected cells is consistent with previous studies in other herpesviruses showing that US3 deletion caused primary enveloped virion accumulation^28,37–40^. When US3 was repaired in the revertant virus, virion accumulation in the nucleus was reduced, and the vesicle-like structure disappeared (Fig. 6g–i), indicating that the DPV US3 protein affected virion nuclear egress, including nucleocapsid egress from the nucleus to form primary enveloped virions and de-envelopment of primary enveloped virions.

Furthermore, a quantitative analysis of virions at different morphogenetic stages was performed using 12 infected cells that were randomly observed under low-magnification electron microscopy. The data presented in Table 1 show the percentages of virions in different morphogenetic stages. In parental virus-and revertant virus-infected DEF cells, 33% and 32% of virions were found in the nucleus, and only 3% and 4% were found in the perinuclear space, respectively. Most virions (64% and 65%) were observed in the cytoplasm and extracellular space. However, virions in the US3-deleted mutant-infected cells were significantly evident in the nucleus (57%) and perinuclear space (7%), with a nearly 2-fold increase compared with the parental and revertant viruses. A small number of virions in the cytoplasm and extracellular space (36%) were found in the US3-deleted mutant-infected cells, with a nearly 2-fold decrease compared with the parental and revertant viruses. All the results indicated that the US3-deleted mutant caused virion accumulation in the nucleus and perinuclear space, and that the mutant had a defect related to virion nuclear egress, which is consistent with the reduced viral titers and plaque sizes described above.

**Table 1:** Virions observed in infected DEF cells by electron microscopy.

Virus	% of virions in different morphogenetic stages (particles in a stage)			Total Counted (Virion /Cells)
	Nucleus		Cytoplasm and Extracellular	
	Intranuclear	Perinuclear area		
BAC-CHv	33% (77)	3% (7)	64% (149)	233/12
BAC-CHv-US3	57% (222)	7% (26)	36% (140)	388/12
BAC-CHv-US3R	32% (68)	4% (8)	65% (139)	215/12

## Discussion

To characterize the impact of the US3 protein on DPV replication, a US3-deleted mutant and the corresponding revertant mutant were constructed. During the rescue of the recombinant viruses, green fluorescent plaques of the US3-deleted mutant were first generated later than those of the revertant virus after transfection (Fig. 2b,c), implying that US3 protein might affect DPV replication. This possibility was verified by the viral growth curve. Regardless of the intracellular or supernatant source, the viral titers of the US3-deleted mutant were significantly lower than those of the parental and revertant viruses from 12 hpi, with an approximately 100-fold reduction observed between 48 and 72 hpi (Fig. 3a,b). The reduced viral titers caused by deletion of US3 were irrelevant to viral DNA generation as the genome copies of the three strains showed no significant differences (Fig. 3c) compared with the viral titers depicted in Fig. 3d. In addition, the efficiency of infectious virion formation of the US3-deleted virus was obviously lower than those of the parental and revertant viruses (Fig. 3e), indicating that US3 protein was important for DPV replication and might play a role in the maturation of infectious virions.

Electron microscopy analysis of DEF cells infected with the US3-deleted mutant revealed a role of the DPV US3 protein in virion nuclear egress. The virion maturation processes of DPV are shown in Fig. 5, and the processes of virion nuclear egress included: nucleocapsids budded at the inner nuclear membrane (INM) to form primary enveloped virions in the perinuclear space, and the primary enveloped virions then fused with the outer nuclear membrane (ONM) to de-envelope and release the nucleocapsids into the cytoplasm for further maturation (Fig. 5c–e). Previous studies have reported that US3 deficiency causes extensive accumulation of primary enveloped virions in the perinuclear space^28,31,37,38,40^, indicating that the process of primary enveloped virion fusion with the ONM to release nucleocapsids into the cytoplasm was blocked. The gB protein is essential for fusion events in herpesviruses due to the phosphorylation and regulation of gB by US3^30,40^. UL34, a transmembrane protein and substrate of US3 protein, may also participate in this process of de-envelopment. However, in our results, although the US3-deleted mutant caused virion accumulation in the perinuclear space, more virions were trapped in the nucleus than in the perinuclear space (Fig. 6d–f), suggesting that US3 deletion might cause stronger blockade of the process of nucleocapsid budding at the INM to form primary enveloped virions. HSV-1 UL31 and UL34 proteins in the form of a heterodimeric complex are the only proteins required for the formation of primary enveloped virions. This complex recruits viral and cellular kinases, such as US3 or UL13, and protein kinase C (PKC) to phosphorylate and soften the nuclear lamina, subsequently allowing nucleocapsids to access the INM^31,41^. UL31 or UL34 deletion blocked virion nuclear egress, and all nucleocapsids accumulated in the nucleus^42,43^. Although UL47 deletion also blocks virion nuclear egress, the researchers suggested that UL47 protein played a regulatory role only in nucleocapsid nuclear egress^29^. Therefore, we propose the following speculations based on our data: (1) The DPV US3 protein affects gB-regulated fusion, while US3 deletion blocks the fusion of primary enveloped virions with the ONM, causing parts of primary enveloped virions to accumulate in the perinuclear space. (2) US3 deletion inhibits softening or local dissolution of the nuclear lamina. Simultaneously, other viral and cellular protein kinases cannot be recruited to phosphorylate the nuclear lamina in DEF cells in a timely manner, leading to another obstacle to form as the process of nucleocapsid budding at the INM is blocked. (3) In HSV-1, the UL31/UL34 complex interacts with the capsid proteins UL17/UL25 to preferentially recruit nucleocapsids into primary enveloped virions. US3 protein interacts with UL31/UL34 and UL47, and UL47 interacts with UL17^29,41^. Therefore, US3, UL31/UL34, UL17/UL25 and UL47 proteins likely form high-ordered complexes. DPV US3 deletion may disturb the formation of this high-ordered complex and inhibit the recruitment of nucleocapsids into primary enveloped virions.

Viruses use two methods to spread to uninfected cells: cell-free infection and cell-to-cell spread. All herpesviruses have a “cell-to-cell” spreading mechanism by which virions enter adjacent cells, facilitating rapid viral dissemination and immune evasion^44^. Previous studies on DPV indicated that UL54 and gJ proteins both played roles in DPV cell-to-cell spread^6,17^. In our results, the DPV US3 protein also affected viral cell-to-cell spread (Fig. 4). The four modes of herpesvirus cell-to-cell spread include: (1) cell-cell fusion, (2) movement across neural synapses, (3) movement across tight junctions, and (4) recruitment of actin-containing structures^45^. Considering other herpesvirus studies, we speculate that the DPV cell-to-cell transmission induced by US3 protein involves the recruitment of actin-containing structures. Many reports have shown that US3 protein induces actin reorganization^26,32,37,38^. In PRV, US3 protein was first reported to cause dramatic alterations in the actin cytoskeleton and to generate long actin- and microtubule-containing cell projections, which allowed virions to move into neighboring cells. This process was related to the phosphorylation of PAKs, the key regulators in the Rho GTPase signaling pathways, by US3^26,32^. In addition, Rho A and Cofilin proteins in the Rho GTPase signaling pathways were also reported to participate in US3-mediated cytoskeletal rearrangements^46,47^.

In summary, in this study, we found that the DPV US3 protein played a pivotal role in viral replication by regulating viral cell-to-cell spread and virion nuclear egress. We hope that this study provides basic information about the DPV US3 protein and a foundation for US3 function research and DPV prevention and control.

## Materials and Methods

### Ethics statement

This study was approved by the Committee of Experiment Operational Guidelines and Animal Welfare of Sichuan Agricultural University (No. XF2014-18). Experiments were conducted in accordance with approved guidelines.

### Cells and viruses

DEF cells were harvested from 9-day-old duck embryos and incubated at 37 °C with 5% CO_2_ in minimal essential medium (MEM; Gibco, Rockford, USA) containing 10% newborn bovine serum (NBS; Gibco, Rockford, USA). The parental virus (BAC-CHv) was obtained from our laboratory.

### Construction of the recombinant pBACs

The whole DPV CHv strain genome (Gene bank: JQ647509.1) with an enhanced green fluorescent protein (EGFP) gene was inserted into a BAC to generate the plasmid of pBAC-CHv, and then pBAC-CHv was transfected into *E. coli* GS1783 in our laboratory^35^. The recombinant pBAC-CHv-ΔUS3 was developed based on a scarless Red recombination system in *E. coli* GS1783^36^. In brief, a linear PCR product in which an I-SceI site, the KanR cassette and a 40-bp sequence duplication were flanked by 40-bp homology arms was amplified, and then the PCR product was electroporated into *E. coli* GS1783 containing pBAC-CHv to induce the first Red recombination through the 40-bp homology arms, resulting in replacement of the US3 gene with the KanR cassette. Subsequently, the KanR cassette was removed by *in vivo* cleavage of the I-SceI site and a second Red recombination through the 40-bp sequence duplication. For the construction of pBAC-CHv-ΔUS3R, nearly identical procedures were carried out, except that the linear PCR product in which the US3 gene, an I-SceI site, the KanR cassette and a 40-bp sequence duplication were flanked by 40-bp homology arms was electroporated into *E. coli* GS1783 containing pBAC-CHv-ΔUS3. All of the primers used in this study are listed in Table 2.

**Table 2:** Primers used in this paper.

Primer name	Sequence 5′-3′	Product
ΔUS3-F	AACAAACATACAAAACTGCCGCGGACGCAGCTCAAATGAATAGGGATAACAGGGTAATCGATTT	KanR
ΔUS3-R	TTTTTTACAAACTTGACTCCTCCCATATATTAATTGTAATTTCATTTGAGCTGCGTCCGCGGCAGTTTTGTATGTTTGTTGCCAGTGTTACAACCAAT	
US3-F	GCGCAACGCCTTAGATTTGC	ΔUS3/ US3R identification product
US3-R	GCGCCAGCTGGTATAACTAC	
RUS3-F	GATTGCTCATATCAAACGAGAACAAACATACAAAACTGCCGCGGACGCAGCTCAAATGAAATGGAAACGTGTCATACCGAT	US3
RUS3-R	ACTCCTCCCATATATTAATTGTAATTTACCCTTTGTGGGTAATAAC	
RUS3-Kan-F	ATTACAATTAATATATGGGAGGAGTCAAGTTTGTAAAAAAGTGTATAAATTAGGGATAACAGGGTAATCGAT	KanR
RUS3-Kan-R	ATCGCCGCAACGCGCATAGCTACTTGTCTAATTTATACACTTTTTTACAAACTTGACTCCTCCCATATATTAATTGTAATTGTTACAACCAATTAACC	

### Rescue and identification of recombinant viruses

The recombinant pBACs were extracted using the QIAGEN Plasmid Midi Kit (QIAGEN, Germany) according to the manufacturer’s recommendations and transfected into DEF cells. DEF cells were cultured and maintained until a large number of green fluorescent plaques were generated. The cells were collected and passed in new DEF cells at least 3 times. The obtained viruses were identified by PCR and Western blotting.

### Determination of viral titers and genome copies in growth kinetics

The procedures were performed as described previously^17,18^. Briefly, DEF cells in 24-well plates were infected with 0.05 MOI of BAC-CHv, BAC-CHv-ΔUS3 or BAC-CHv-ΔUS3R. Samples of the infected cells and their supernatants were collected separately at 12, 24, 48, and 72 hpi. The volume of each sample was increased to 500 µl with MEM, and then the samples were frozen and thawed 3 times. Intracellular viral titers and supernatant viral titers were detected by determining the 50% tissue culture infectious dose (TCID_50_). All experiments were repeated 3 times.

Viral genome copies were detected by qPCR. Viral DNA of total cells was extracted using the Magen HiPure Viral DNA Kit (Magen, Guangzhou, China). Premix Ex Taq™ (Probe qPCR) (Takara, Dalian, China) was used to determine viral genome copies. The primers and probe used to detect the BAC-CHv UL30 gene by qPCR were designed previously in our laboratory. PCR amplifications were performed under the following conditions: 95 °C for 30 s, followed by 40 cycles at 95 °C for 5 s and 60 °C for 30 s. Then, the PCR products were quantified by comparison with the established standard curve of the laboratory. All experiments were repeated 3 times.

### Plaque morphology of the recombinant viruses

DEF cells in 6-well plates were infected with 0.001 MOI of BAC-CHv, BAC-CHv-ΔUS3 or BAC-CHv-ΔUS3R. After incubation at 37 °C for 2 h, 1% methylcellulose (Solarbio, Beijing, China) was added to cover the cells. At 60 hpi, the cells were observed under a fluorescence microscope (Nikon TI-SR, Japan). Twenty randomly selected green fluorescent plaques were photographed for each strain, and the average plaque sizes were measured using Image-Pro Plus software (Bio-Rad, California, USA)^17^. The plaque sizes were calculated and compared to those of the parental virus, which were set to 100%. In addition, viral plaques of the three strains were also stained with 1.5% crystal violet at 6 dpi, fixed with 4% paraformaldehyde, and imaged using ChemiDoc MP.

### Electron microscopy analysis of recombinant viruses

DEF cells in 6-well plates were infected with 5 MOI of BAC-CHv, BAC-CHv-ΔUS3 or BAC-CHv-ΔUS3R. At 20 hpi, the cells were collected by scraping, centrifuged at 3,000 rpm for 10 min, and fixed with 2.5% glutaraldehyde at 4 °C overnight. All samples were then sent to the Harbin Veterinary Research Institute (Harbin, China) for analysis under a transmission electron microscope (Hitachi H-7650, Tokyo, Japan).

## Supplementary information

Supplementary Information.

## References

[1] Cheng, A. Duck plague (ed. Cheng A.) 1–4 (Beijing, 2015).

[2] Ying W (2012). Complete genomic sequence of Chinese virulent duck enteritis virus. Journal of Virology.

[3] Ying W (2012). Comparative genomic analysis of duck enteritis virus strains. Journal of Virology.

[4] Liu C (2015). Duck enteritis virus UL54 is an IE protein primarily located in the nucleus. Virology Journal.

[5] Liu C (2016). Characterization of nucleocytoplasmic shuttling and intracellular localization signals in Duck Enteritis Virus UL54. Biochimie.

[6] Liu C (2017). Regulation of viral gene expression by duck enteritis virus UL54. Scientific Reports.

[7] Gao X (2017). Duck enteritis virus (DEV) UL54 protein, a novel partner, interacts with DEV UL24 protein. Virology Journal.

[8] Hu, X. *et al*. The duck enteritis virus early protein, UL13, found in both nucleus and cytoplasm, influences viral replication in cell culture. Poultry Science 96, 10.3382/ps/pex043 (2017). 10.3382/ps/pex043 28371814

[9] Gao J (2015). Identification and characterization of the duck enteritis virus (DEV) US2 gene. Genetics & Molecular Research Gmr.

[10] Zhao C (2019). Molecular characterization and antiapoptotic function analysis of the duck plague virus Us5 gene. Scientific Reports.

[11] Zhang D (2017). Molecular characterization of the duck enteritis virus US10 protein. Virology Journal.

[12] He Q (2012). Replication kinetics of duck enteritis virus UL16 gene *in vitro*. Virology Journal.

[13] Ming-Sheng C (2010). Characterization of the duck plague virus UL35 gene. Intervirology.

[14] He T (2018). Molecular characterization of duck enteritis virus UL41 protein. Virology Journal.

[15] Zhang S (2011). Characterization of duck enteritis virus UL53 gene and glycoprotein K. Virology Journal.

[16] Wu Y (2011). Establishment of real-time quantitative reverse transcription polymerase chain reaction assay for transcriptional analysis of duck enteritis virus UL55 gene. Virology Journal.

[17] You Y (2018). Duck plague virus Glycoprotein J is functional but slightly impaired in viral replication and cell-to-cell spread. Scientific Reports.

[18] Ma Y, Zeng Q, Wang M, Cheng A, Chen X (2018). US10 Protein Is Crucial but not Indispensable for Duck Enteritis Virus Infection *in Vitro*. Scientific Reports.

[19] Wu Y (2017). Preliminary study of the UL55 gene based on infectious Chinese virulent duck enteritis virus bacterial artificial chromosome clone. Virology Journal.

[20] Akihisa K (2005). Identification of proteins phosphorylated directly by the Us3 protein kinase encoded by herpes simplex virus 1. Journal of Virology.

[21] Akihisa K (2011). Herpes simplex virus 1 protein kinase Us3 and major tegument protein UL47 reciprocally regulate their subcellular localization in infected cells. Journal of Virology.

[22] Kato A (2009). Herpes simplex virus 1 protein kinase Us3 phosphorylates viral envelope glycoprotein B and regulates its expression on the cell surface. Journal of Virology.

[23] Mou F, Forest T, Baines JD (2007). US3 of herpes simplex virus type 1 encodes a promiscuous protein kinase that phosphorylates and alters localization of lamin A/C in infected cells. Journal of Virology.

[24] Wang K, Ni L, Wang S, Zheng C (2014). Herpes Simplex Virus 1 Protein Kinase US3 Hyperphosphorylates p65/RelA and Dampens NF-κB Activation. Journal of Virology.

[25] Shuai W, Kezhen W, Rongtuan L, Chunfu Z (2013). Herpes Simplex Virus 1 Serine/Threonine Kinase US3 Hyperphosphorylates IRF3 and Inhibits Beta Interferon Production. Journal of Virology.

[26] Céline VDB (2009). Alphaherpesvirus US3-mediated reorganization of the actin cytoskeleton is mediated by group A p21-activated kinases. Proceedings of the National Academy of Sciences of the United States of America.

[27] Ching-Dong C (2013). Suppression of apoptosis by pseudorabies virus Us3 protein kinase through the activation of PI3-K/Akt and NF-κB pathways. Research in Veterinary Science.

[28] Reynolds AE, Wills EG, Roller RJ, Ryckman BJ, Baines JD (2002). Ultrastructural localization of the herpes simplex virus type 1 UL31, UL34, and US3 proteins suggests specific roles in primary envelopment and egress of nucleocapsids. Journal of Virology.

[29] Zhuoming L (2014). Herpes simplex virus 1 UL47 interacts with viral nuclear egress factors UL31, UL34, and Us3 and regulates viral nuclear egress. Journal of Virology.

[30] Takahiko I, Ken S, Jun A, Yasushi K (2010). Effects of phosphorylation of herpes simplex virus 1 envelope glycoprotein B by Us3 kinase *in vivo* and *in vitro*. Journal of Virology.

[31] Mou F, Wills EG, Park R, Baines JD (2008). Effects of lamin A/C, lamin B1, and viral US3 kinase activity on viral infectivity, virion egress, and the targeting of herpes simplex virus U(L)34-encoded protein to the inner nuclear membrane. Journal of Virology.

[32] Favoreel HW, Minnebruggen GV, Adriaensen D, Nauwynck HJ (2005). Cytoskeletal rearrangements and cell extensions induced by the US3 kinase of an alphaherpesvirus are associated with enhanced spread. Proceedings of the National Academy of Sciences of the United States of America.

[33] Deng L (2018). Suppression of NF-κB Activity: A Viral Immune Evasion Mechanism. Viruses.

[34] You Y (2017). The suppression of apoptosis by α-herpesvirus. Cell Death & Disease.

[35] Wu, Y. Genome analysis of duck plague virus Chinese virulent strain and preliminary study of UL55 gene fuction. Sichuan Agricultural University (2015).

[36] Tischer BK, Smith GA, Osterrieder N (2010). En passant mutagenesis: a two step markerless red recombination system. Methods Mol Biol.

[37] Daniel S, Caleb MK, Kaufer BB, Nikolaus O (2008). Enzymatically inactive U(S)3 protein kinase of Marek’s disease virus (MDV) is capable of depolymerizing F-actin but results in accumulation of virions in perinuclear invaginations and reduced virus growth. Virology.

[38] Proft A (2016). The Role of the Equine Herpesvirus Type 1 (EHV-1) US3-Encoded Protein Kinase in Actin Reorganization and Nuclear Egress. Viruses.

[39] Wisner TW (2009). Herpesvirus gB-Induced Fusion between the Virion Envelope and Outer Nuclear Membrane during Virus Egress Is Regulated by the Viral US3 Kinase. Journal of Virology.

[40] Wisner TW (2009). Herpesvirus gB-induced fusion between the virion envelope and outer nuclear membrane during virus egress is regulated by the viral US3 kinase. Journal of Virology.

[41] Mettenleiter TC, Müller F, Granzow H, Klupp BG (2013). The way out: what we know and do not know about herpesvirus nuclear egress. Cellular Microbiology.

[42] Roller RJ, Zhou Y, Schnetzer R, Ferguson J, Desalvo D (2000). Herpes simplex virus type 1 U(L)34 gene product is required for viral envelopment. Journal of Virology.

[43] Chang YE (1997). The null mutant of the U(L)31 gene of herpes simplex virus 1: construction and phenotype in infected cells. Journal of Virology.

[44] Carmichael JC, Yokota H, Craven RC, Schmitt A, Wills JW (2018). The HSV-1 mechanisms of cell-to-cell spread and fusion are critically dependent on host PTP1B. Plos Pathogens.

[45] Quentin S (2008). Avoiding the void: cell-to-cell spread of human viruses. Nature Reviews Microbiology.

[46] Thary J, Céline VDB, Cliff VW, Leen VT, Favoreel HW (2015). Pseudorabies virus US3 triggers RhoA phosphorylation to reorganize the actin cytoskeleton. Journal of General Virology.

[47] Thary J (2013). Alphaherpesviral US3 kinase induces cofilin dephosphorylation to reorganize the actin cytoskeleton. Journal of Virology.

