# Supplementary material for: The Pivotal Roles of US3 Protein in Cell-to-Cell Spread and Virion Nuclear Egress of Duck Plague Virus
Source: Sci Rep. 2020 Apr 28;10:7181. doi: 10.1038/s41598-020-64190-2 (PMC7189242; doi:10.1038/s41598-020-64190-2)

The Pivotal Roles of US3 Protein in Cell-to-Cell Spread and Virion Nuclear Egress of Duck Plague Virus

Liyao Deng[1,2,3†], Mingshu Wang[1,2,3†*], Anchun Cheng[1,2,3*], Qiao Yang[1,2,3], Ying Wu[1,2,3], Renyong Jia[1,2,3], Shun Chen[1,2,3], Dekang Zhu[2,3], Mafeng Liu[1,2,3], Xinxin Zhao[1,2,3], Shaqiu Zhang[1,2,3], Juan Huang[1,2,3], Xumin Ou[1,2,3], Sai Mao[1,2,3], Ling Zhang[1,2,3], Yunya Liu[1,2,3], Yanling Yu[1,2,3], Bin Tian[1,3], Leichang Pan[1,3], Mujeeb Ur Rehman[1,3] and Xiaoyue Chen[2,3]

[1]Institute of Preventive Veterinary Medicine, Sichuan Agricultural University, Wenjiang 611130, People's Republic of China.

[2]Avian Disease Research Center, College of Veterinary Medicine of Sichuan Agricultural University, Wenjiang 611130, People's Republic of China.

[3]Key Laboratory of Animal Disease and Human Health of Sichuan Province, Sichuan Agricultural University, Wenjiang 611130, People's Republic of China.

* Correspondence: chenganchun@vip.163.com, mshwang@163.com

† These authors contributed equally to this work

Fig. 2a. Identification of recombinant pBACs by PCR analysis.

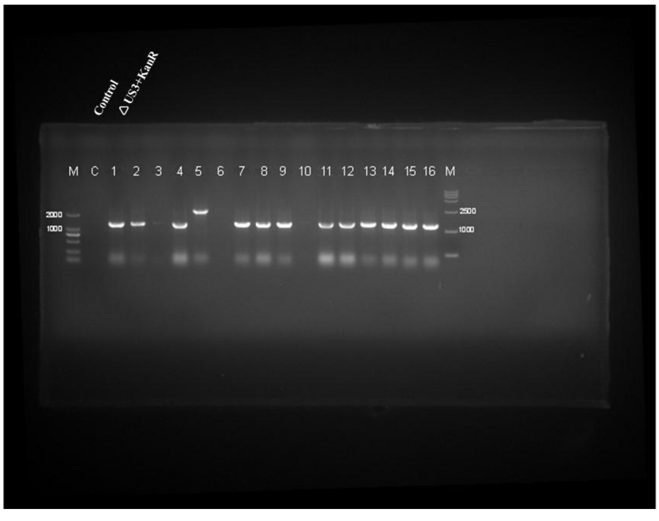

Identification of pBAC-CHv-△US3 (Lane 1 and 2)

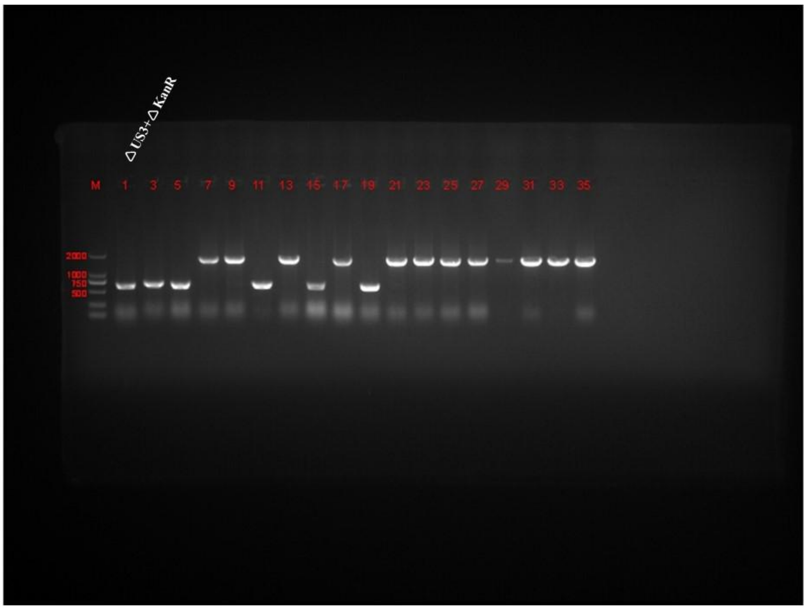

Identification of pBAC-CHv-△US3 (Lane 3)

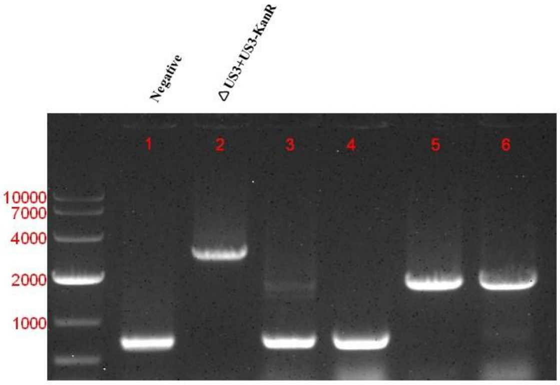

Identification of pBAC-CHv-△US3R (Lane 4 and 5)

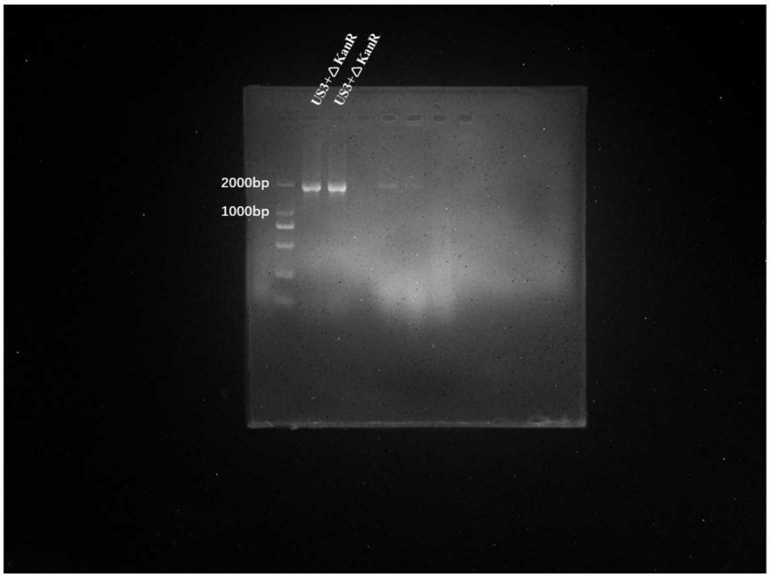

Identification of pBAC-CHv-△US3R (Lane 6)

Fig. 2c. The observed DEF cells at 6 d after pBAC-CHv-△US3R transfection.

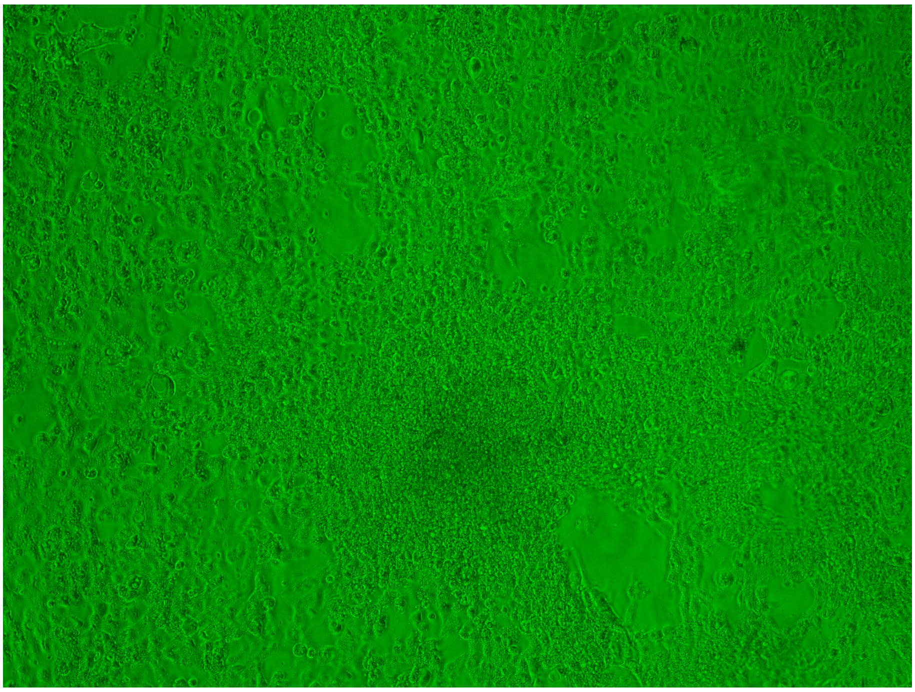

Fig. 2d. Identification of recombinant viruses by PCR analysis.

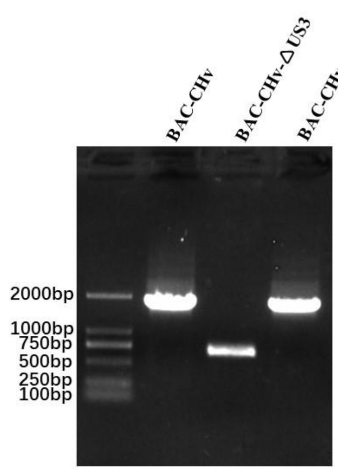

Fig. 2e. Western blot analysis of US3 protein expression of recombinant viruses.

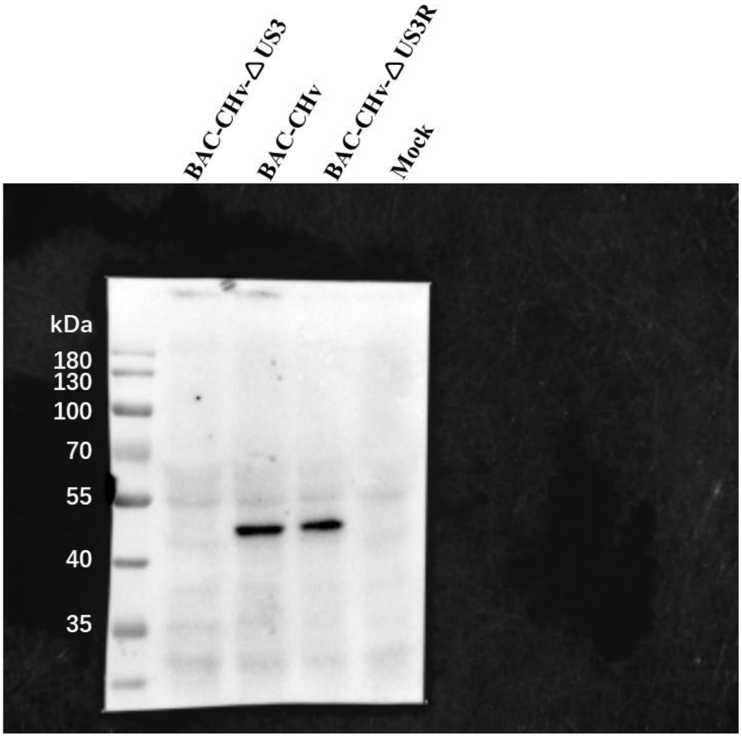

The protein expression of US3 (50kDa)

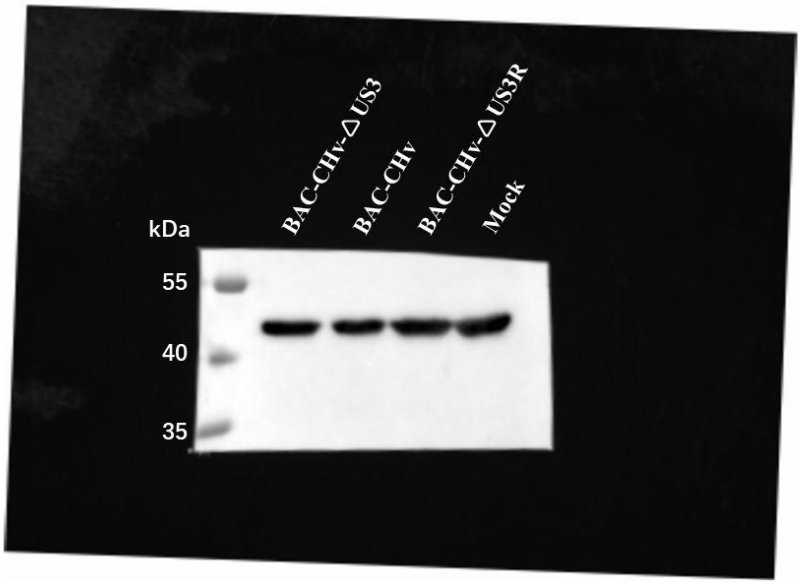

The protein expression of β-actin (42kDa, as a control)

Fig. 4d. Images of viral plaques after 1.5% crystal violet staining.

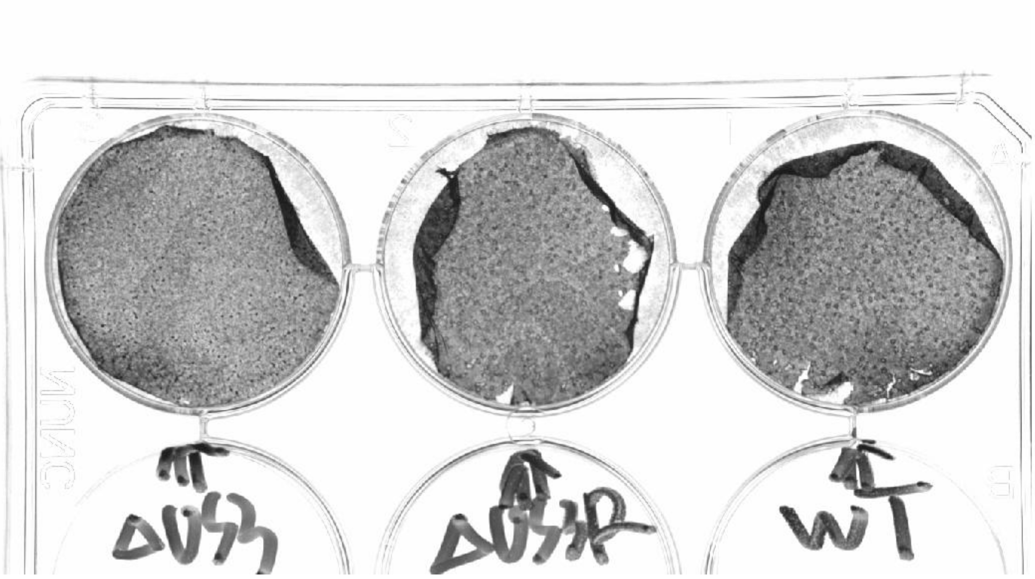

Supplement: Supplementary file 1 — Supplementary Information. [file 41598_2020_64190_MOESM1_ESM.pdf]
